# Bilateral posterior RION after concomitant radiochemotherapy with temozolomide in a patient with glioblastoma multiforme: a case report

**DOI:** 10.1186/1471-2407-10-520

**Published:** 2010-10-01

**Authors:** Stefanie Schreiber, Vanessa Prox-Vagedes, Erck Elolf, Ines Brueggemann, Guenther Gademann, Imke Galazky, Claudius Bartels

**Affiliations:** 1 Department of Neurology, Otto-von-Guericke University, Leipziger Straße 44, 39120 Magdeburg, Germany; 2 Department of Psychiatry, Social Psychiatry und Psychotherapy, Hannover Medical School, Carl-Neuberg-Straße 1, 30625 Hannover, Germany; 3 Department of Neuroradiology, Otto-von-Guericke University, Leipziger Straße 44, 39120 Magdeburg, Germany; 4 Department of Radiation Therapy, Otto-von-Guericke University, Leipziger Straße 44, 39120 Magdeburg, Germany

## Abstract

**Background:**

Radiation induced optic neuropathy (RION) is a rare but severe consequence of radiation therapy that is associated with adjuvant chemotherapy, specifically therapy with vincristine or nitrosoureas. However, there is very little evidence regarding the occurrence of RION after concomitant radiochemotherapy with temozolomide.

**Case Presentation:**

The case of a 63 year old woman with glioblastoma multiforme and concomitant radiochemotherapy with temozolomide is described. Due to a slight depressive episode the patient also took hypericum perforatum. Five months after cessation of fractionated radiation and adjuvant chemotherapy with temozolomide (cumulative dose of 11040 mg) the patient developed bilateral amaurosis due to RION. Tumor regrowth was excluded by magnetic resonance imaging. After the application of gadolinium a pathognomonic contrast enhancement of both prechiasmatic optic nerves could be observed.

**Conclusions:**

In this patient, the occurrence of RION may have been the result of radiosensitization by temozolomide, which could have been strengthened by hypericin. Consequently, physicians should avoid a concomitant application of hypericum perforatum and radiochemotherapy.

## Background

Radiation-induced optic neuropathy (RION) is a complication of radiotherapy to the anterior visual pathway. It can occur from 3 months to more than 8 years after radiation exposure [1]. Latency to the onset of symptoms is related to the radiation dose: A total cumulative dose of fractionated radiation above 63 Gy is associated with an increasing risk inducing an optic neuropathy [2]. Additional predisposing risk factors are age and diabetes mellitus [2]. In addition, a pre-existing compression to the optic nerves and the chiasm may predispose these structures to injury by radiotherapy [3]. RION has been described mainly in patients with tumors of nasopharynx, paranasal sinuses, nasal cavity, pituitary adenoma, craniopharyngioma, skull base and orbita [2,4,5]. Since some chemotherapeutic agents, namely vincristine, nitrosoureas and cisplatin [6,7] are associated with optic nerve toxicity and/or can serve as radiosensitizers [8], those patients receiving adjuvant chemotherapy have an especially high risk of developing RION.

To our knowledge, there is only one glioblastoma patient in literature who developed a left-sided optic neuropathy after concomitant radiochemotherapy with temozolomide and bevacizumab [9]. However, since there was a damage of the optic pathway as a secondary consequence of tumor growth a relation to the applied radiochemotherapy remained doubtful.

Therefore, although there is substantial literature concerned with RION, a bilateral radiation induced optic neuropathy has never been described in a patient with glioblastoma multiforme who was concomitantly treated only with temozolomide. Here, we present such a case.

## Case presentation

**Figure 1:**
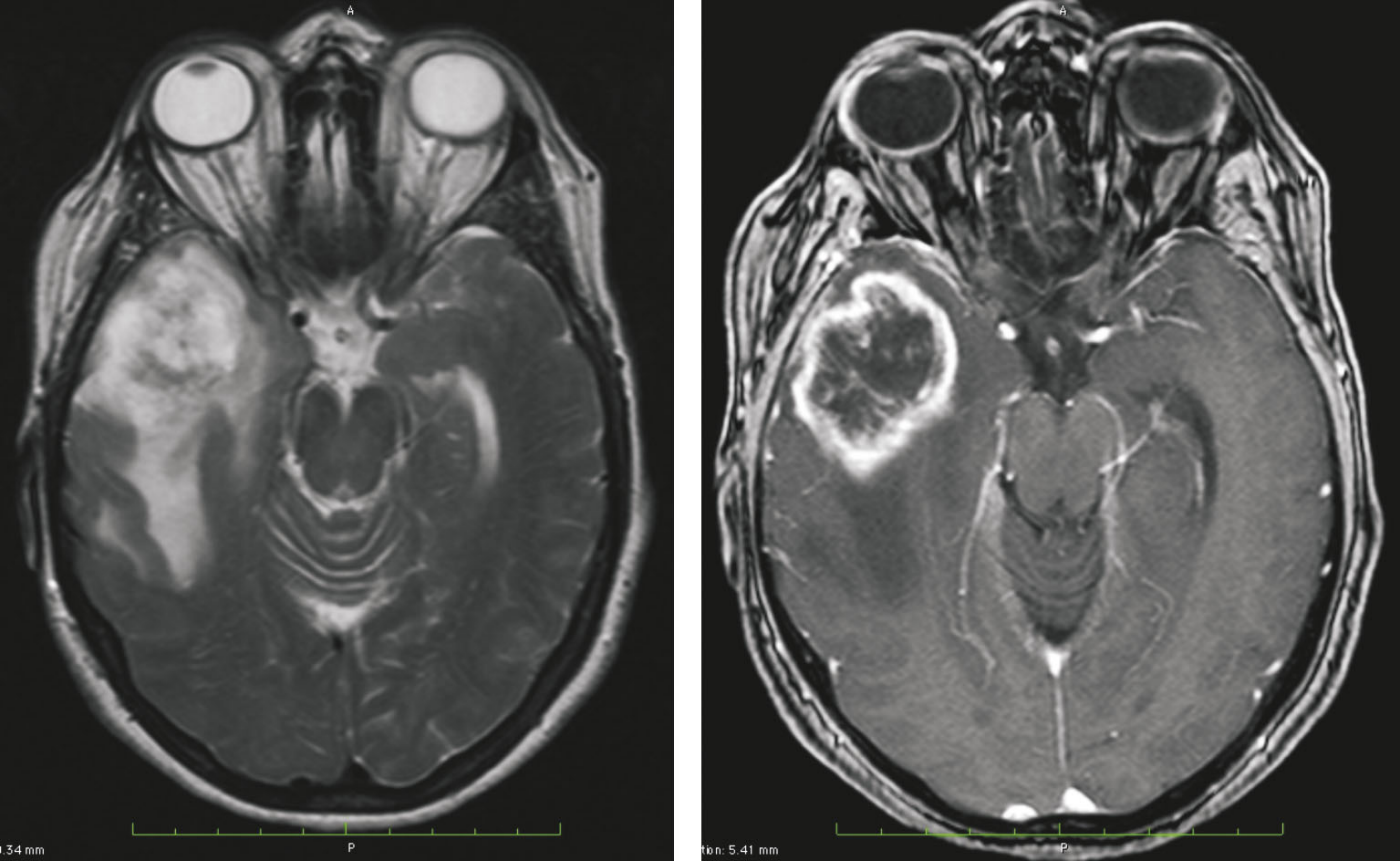
**Images before surgery and radiochemotherapy**. On T2 (Figure 1A) and gadolinium enhanced T1 (Figure 1B) weighted magnetic resonance imaging in histologically confirmed glioblastoma multiforme of the right temporal lobe can be seen. Note that there is no compression/infiltration of the adjacent optic nerve, the chiasm or the right internal carotid artery.

**Figure 2:**
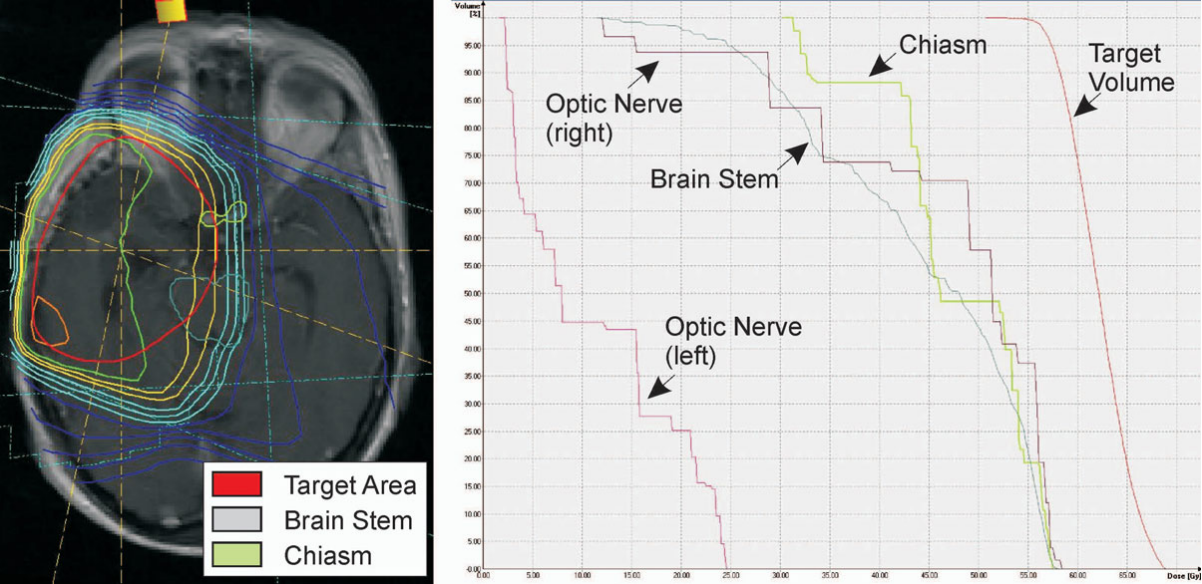
**Target volume and dose-volume histogram**. Figure 2A Magnetic resonance imaging after open surgery with isodose lines from 10 to 110%. Target volume is marked in red, chiasm in green and brainstem in blue. Figure 2B Dose-volume histogram (DVH) of the radiation shows the dose given to the percentage of OAR (organ at risk) volume, i.e. less than 20% of the chiasm was irradiated with 56 Gy within 6 weeks.

A 63 year old woman was presented with severe headache and psychomotor slowing that had recurred for some weeks. Her medical history revealed a mild arterial hypertension and a slight depressive episode with sleep disturbances. To treat the depressive episode the patient self-medicated with hypericin (St. John's wort, hypericum perforatum) with a dosage of 900 mg per day, probably for several months before she was transferred to our department. No vision disorders were found. MRI revealed a contrast-enhanced 4.6 × 3.8 × 3.1 cm lesion in her right temporal lobe, with no observable compression of the optic nerves or optic chiasm (Figure 1A, B). A glioblastoma multiforme was suspected and confirmed histologically after open surgery with a resection of more than 90% of tumor tissue. After surgery, concurrent photon radiation (involved field, two co-axial isocentric fields, fractionated, once daily; cumulative dose 60 Gy, fraction size 2 Gy) and chemotherapy with temozolomide (75 mg/m^2 ^body surface, total dose of 5040 mg) were implemented for a duration of 6 weeks. Target volumes and organs at risk, such as chiasm, were delineated on MRI (Figure 2A). The maximum doses to the optic nerves and chiasm were obtained from dose-volume histograms (DVHs) (Figure 2B). The maximal cumulative radiation dose of 56 Gy (maximal single dose 1.8 Gy) was delivered to less than 20% of the optic chiasm. Residual parts of the optic decussation were irradiated with a median cumulative dose of 46 Gy. Maximal cumulative dose to the right and left optic nerves were 57 Gy and 24 Gy. After starting radiation, the patient described a headache that also affected the right eye, with symptoms occurring daily and increasing during the course of the day. Karnofsky performance status [10] remained stable at about 70%. Six weeks after completion of the initial course of treatment (concomitant radiochemotherapy), 4 additional cycles of chemotherapy with temozolomide (150-200 mg/m^2 ^body surface, total dose of 6000 mg) were performed.

**Figure 3:**
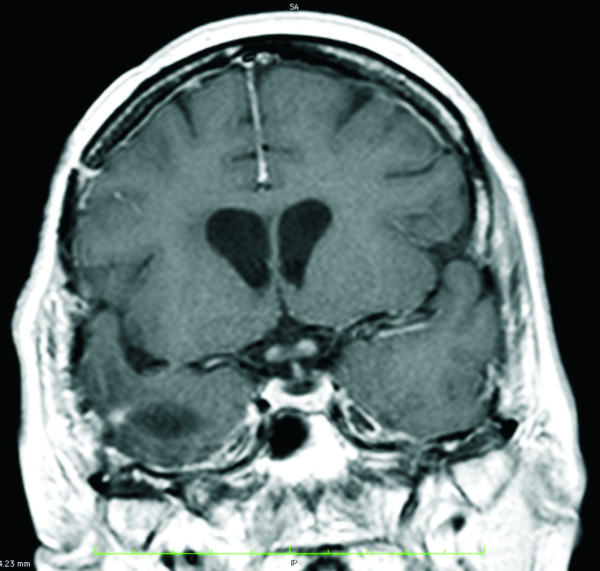
**Images 5 months after cessation of radiation**. T1 weighted gadolinium enhanced magnetic resonance imaging revealing subtle contrast enhancement of the left and right prechiasmatic optic nerve 5 months after cessation of radiation when bilateral amaurosis occurred. Note that there is no infiltration of the chiasm by tumor recurrence.

A progressive visual loss due to bilateral amaurosis occurred 5 months after cessation of radiation. This symptom was accompanied by visual hallucinations. Ophthalmological examination revealed bilateral amaurotic fixed pupils and a slightly pale optic disc. Vision loss from other causes, such as optic nerve or chiasm compression by tumor progression (MRI [Figure 3] and single photon emission computed tomography [SPECT] were performed), retinal disorders, cataract, or bilateral giant cell arteritis, were carefully excluded. Detected visual acuity was 0/100 in both eyes. No visual evoked potentials could be generated by either monocular or binocular excitation with flashing lights and contrast inversions. Except for a slight elevation of protein concentration, there were no indications of infectious disease or other pathological findings in the patient's cerebrospinal fluid. Four months after RION appeared the visual loss persisted; Karnofsky performance status declined to 40%.

## Conclusions

Here we present the first report of a patient with glioblastoma multiforme developing a bilateral RION about 5 months after cessation of concomitant radiochemotherapy with temozolomide. An intrinsic mass in the optic nerves and/or chiasm was excluded by MRI, as was a compression of those structures by a possible recurrence of the tumor. In addition, a contrast enhancement of both prechiasmatic optic nerves could be observed after gadolinium application (Figure 3). Gadolinium enhancement of the optic nerve anywhere from its extraocular-intraorbital part to the optic chiasm seems to be a characteristic finding of RION in MR-Imaging, since it has been described by several groups as the most common type of radiation induced optic neuropathy [11,12]. Accumulation of the contrast medium could be explained by a disruption of the blood-brain-barrier [5] that may play a role in the pathogenesis of RION, which is associated with delayed radionecrosis, damage and depletion of vascular endothelium and ischemic demyelination [13].

Concerning fractionated once-daily radiation, the 10-year rates for freedom from optic neuropathy are respectively about 96% and 78% after the application of a total optic nerve dose of ≤ 63 Gy and a fraction dose of ≤ 1.8 Gy [2]. Our patient received a total cumulative dose of 60 Gy with a fraction size of 2 Gy and a maximal chiasm and optic nerve dose of 56 Gy and 57 Gy, with less than 20% of the optic chiasm and nerves subjected to that total cumulative dose (Figure 2B). Since prescriptive limits for radiation dose were maintained, other causes that could have aggravated the radiation effect should be taken into consideration. At time of radiation the patient's age was older than 60 years, which has been depicted as significant covariate for vision loss. The patient did not suffer from diabetes, the second significant covariate for RION [4]. Female gender and arterial hypertension have not been found to be significant risk factors for radiation-induced optic neuropathy [4].

In addition, the possible effects of the patients' concomitant therapy with temozolomide and hypericin should be addressed. Interestingly, at least tumor cells undergo apoptosis when treated with photodynamic therapy sensitized with hypericin [14]. This process has been described as being associated with a cleavage of poly-[ADP-ribose]-polymerase (PARP) [15], participating in cellular recovery from DNA damage [16]. An inhibition or cleavage of PARP might enhance the cytotoxicity of temozolomide [17] and might lead to radiosensitization [18] at least in the case of tumorcells. To what extent these processes also take place in the healthy tissue of the optic nerves and the chiasm has not been determined. However, since temozolomide is known to induce at least unilateral visual loss (summary of product characteristics by the producer of temozolomide, Essex Chemie AG, Luzern, Switzerland), produces visual loss when combined with thalidomide [19], serves as a "radiosensitizer" [8], and distributes in normal brain tissue [20], a damaging effect of temozolomide on the optic nerves/chiasm (possibly strengthened by hypericin) is conceivable.

In addition hypericin has inhibitory effects on protein kinase C (PKC) [21], which might be associated with the activation of caspases, required for radiation mediated apoptosis [21]. Hence, several preclinical studies have demonstrated that the inhibition of PKC enhances the effect of ionization radiation [22,23]. On the other hand, tissue warming caused by radiation, accelerates apoptotic effects of hypericin [24]. Thus, an additional interplay between radiation and hypericin, associated with apoptosis of endothelial cells and oligodendrocytes [25], may have occurred.

Since many patients with high-grade glioma suffer from depression, fatigue and sleep disorders [26] and since mild depressive disorders are an indication for the use of St. John's wort [27], many patients with glioblastoma multiforme may take hypericin. In view of the case of bilateral RION we report, and the aforementioned possible connection between St. John's wort and both temozolomide and the effects of radiation, physicians should be careful about administering hypericin simultaneously with radiochemotherapy.

## Competing interests

The authors declare that they have no competing interests.

## Authors' contributions

SS, VP and CB wrote the bulk of manuscript text and managed literature searches; SS is the corresponding author of the manuscript. IB and IG collected patients' data and documented the clinical progression. EE and GG allocated and described the MRI as well as the Dose-volume histogram. All authors read and approved the final manuscript.

## Consent

Written informed consent could not be obtained from the patient for publication of this case report and any accompanying images because the patient has died. The patient's son has consented to publishing the data.

## Pre-publication history

The pre-publication history for this paper can be accessed here:

http://www.biomedcentral.com/1471-2407/10/520/prepub
